# Pleural effusion volume in patients with acute pancreatitis: a retrospective study from three acute pancreatitis centers

**DOI:** 10.1080/07853890.2021.1998594

**Published:** 2021-11-02

**Authors:** Gaowu Yan, Hongwei Li, Anup Bhetuwal, Morgan A. McClure, Yongmei Li, Guoqing Yang, Yong Li, Linwei Zhao, Xiaoping Fan

**Affiliations:** a Department of Radiology, The First Affiliated Hospital of Chongqing Medical University, Chongqing, China; b Department of Radiology, Suining Central Hospital, Suining, China; c Department of Radiology, The Third Hospital of Mianyang, Sichuan Mental Health Center, Mianyang, China; d Sichuan Key Laboratory of Medical Imaging, Department of Radiology, Affiliated Hospital of North Sichuan Medical College, Nanchong, China; e Department of Radiology and Imaging, Institute of Rehabilitation and Development of Brain Function, The Second Clinical Medical College of North Sichuan Medical College, Nanchong Central Hospital, Nanchong, China

**Keywords:** Pleural effusion, computed tomography, acute pancreatitis

## Abstract

**Objective:**

To assess the value of pleural effusion volume (PEV) quantified on chest computed tomography (CT) in patients with early stage acute pancreatitis (AP).

**Methods:**

Data of PEV, and C-reactive protein (CRP) levels as well as Ranson, bedside index of severity in acute pancreatitis (BISAP), Marshall, acute physiology and chronic health evaluation II (APACHE II), CT severity index (CTSI), and extra-pancreatic inflammation on computed tomography (EPIC) scores in patients with AP were collected. Duration of hospitalization, severity of AP, infection, procedure, intensive care unit (ICU) admission, organ failure, or death were included as the outcome parameters.

**Results:**

In 465 patients, the mean PEV was 98.8 ± 113.2 mL. PEV showed strong and significant correlations with the CRP levels, duration of hospitalization as well as the Ranson, BISAP, Marshall, APACHE II, CTSI, and EPIC scores (*p* < .05). PEV demonstrated significant accuracy in predicting severity, infection, procedure, ICU admission, organ failure, and death (*p* < .05).

**Conclusion:**

PEV quantified on chest CT positively associated with the duration of hospitalization, CRP levels, Ranson, BISAP, Marshall, APACHE II, CTSI, and EPIC scores. It can be a reliable radiologic biomarker in predicting severity and clinical outcomes of AP.

**KEY MESSAGES**
- Pleural effusion is a common chest finding in patients with acute pancreatitis.
- Pleural effusion volume quantified on chest CT examination positively associated with the duration of hospitalization, CRP level, as well as Ranson, BISAP, Marshall, APACHE II, CTSI, and EPIC scoring systems.
- Pleural effusion volume can be a reliable radiologic biomarker in the prediction of severity and clinical outcomes of acute pancreatitis.

## Introduction

Acute pancreatitis (AP) is one of the most common acute abdominal diseases in the clinical practice [1,2]. It can be induced by various causes with the initial event being the activation of the pancreatic enzyme within the pancreas. This will then progress to the pancreas itself and its adjacent tissues to be affected with edoema, haemorrhage and even necrosis [3,4]. Among the aetiologies of AP, gallstones, excessive alcohol consumption, hypertriglyceridaemia, and post- endoscopic retrograde cholangiopancreatography (post-ERCP) are the top four [5,6]. In addition, because of an ageing population and the increment in the prevalence of gallstones and obesity, there has been a rise in the incidence of AP all over the world [6,7].

In 1992, a widely accepted Atlanta classification divided AP into two subtypes, i.e. mild AP (associated with minimal organ dysfunction and an uneventful recovery lacking the features of severe pancreatitis) and severe AP (associated with organ failure and/or local complications, such as necrosis, abscess, or pseudocyst) [8]. However, since it was introduced, confusion and inaccurate terminology has existed both in scientific research and in clinical fields [9–14].

With the research progress made over the past 20 years, a revised Atlanta classification was introduced in 2012 in which AP was distinctly divided into acute mild, moderately severe, and severe subtypes [9]. In the 2012 Revised Atlanta Classification (RAC), mild AP is absence of organ failure and local or systemic complications; moderately severe AP is associated with transient organ failure (<48 h), and/or local or systemic complications without persistent organ failure (more than 48 h); while severe AP is characterized by persistent single and/or multiple organ failure (more than 48 h) [9–14]. Mild AP is often self-limiting with very low mortality; moderately severe AP may resolve with or without some interventions; and the mortality is far less than that of severe AP. The severe AP carries a mortality rate of 36–50%, and the patients with infections may suffer from the highest mortality [9–14]. As a result, it is of great importance to inform the severity of AP for the patients and clinicians.

Clinically, CT plays an important role in the diagnosis and management of AP. Previous and recent studies have reported that the extra pancreatic necrosis volume (EPNV) and pancreatic necrosis volume (PNV) both can be good radiological parameters that are highly associated with AP [15–18]. Accordingly, the authors postulate that the prognosis of AP might be correlated with the various volume of pleural effusion quantified on chest CT images. Therefore, we aimed to evaluate whether quantification of pleural effusion volume (PEV), acquired in early CT examinations (1–7 days), is a valuable biomarker in determining the prognosis of AP with a multi centred study from three teaching hospitals.

## Materials and methods

This study was conducted in three medical centres which was approved by the institutional review committee of the Suining Central Hospital, Suining, Sichuan, China (permission number: ynkt-2019125). Owing to the fact that this retrospective nature of this study would not do any harm to the patients concerned, informed consent was waved.

### Patients

Patients admitted and treated in the three medical centres from January 2018 to June 2020 with a confirmed diagnosis of AP were all enrolled into this study. AP was diagnosed according to two of the following three 2012 RAC criteria: (1) abdominal pain in accordance with AP (acute onset of a persistent and severe epigastric pain, often radiating to the back); (2) serum lipase or amylase activity at least three times more than the upper limit of normal level; and (3) characteristic findings of AP on medical imaging [transabdominal ultrasonography, contrast-enhanced CT or magnetic resonance imaging (MRI)] [9–14].

**Figure 1.**
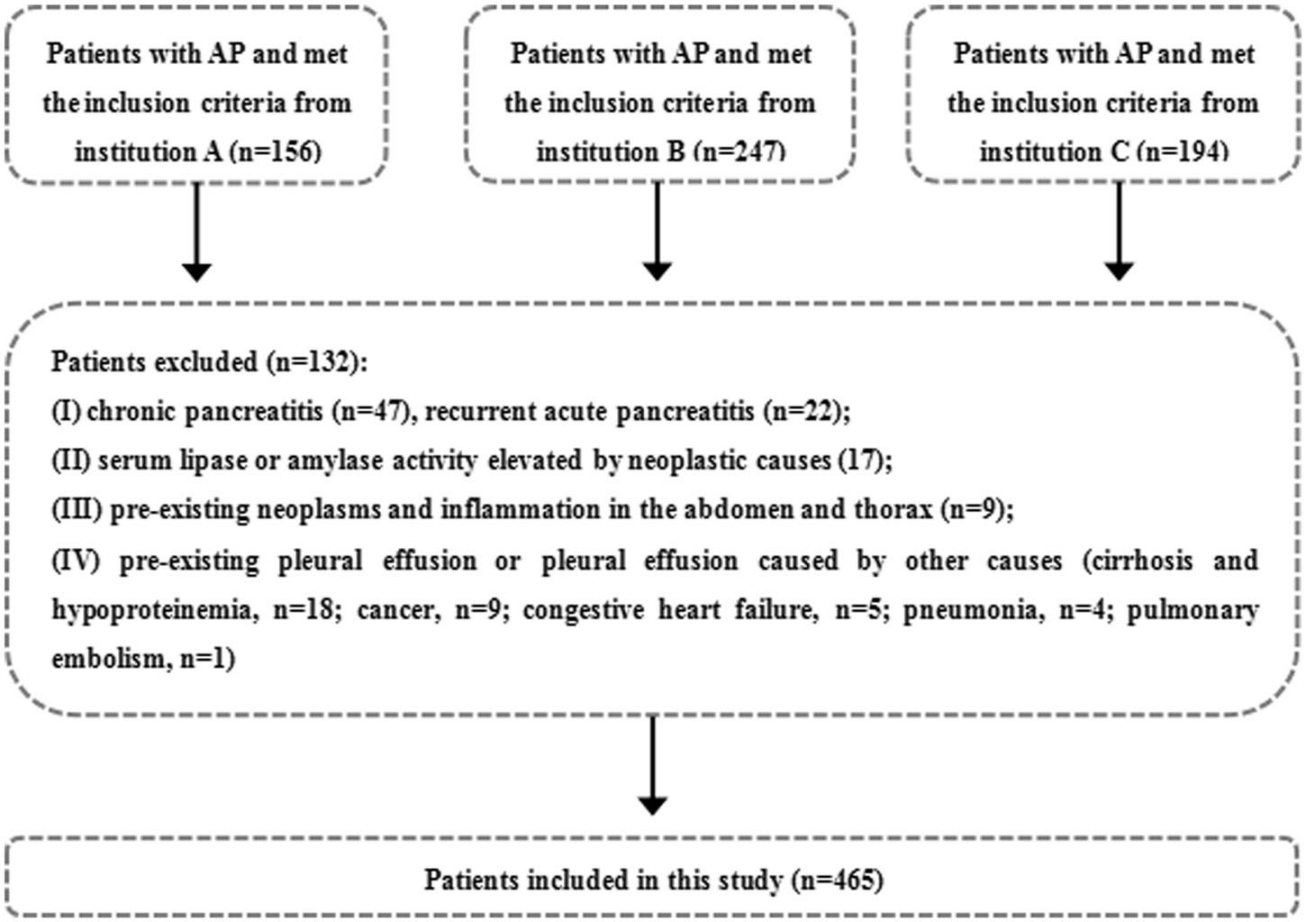
Flow chart for inclusion and exclusion of patients.

The inclusion criteria were: (1) first attack of AP; (2) AP at an early phase (within the 1st week); (3) both the thoracic (non-enhanced) and abdominal (non-enhanced and contrast enhanced) CT examinations were performed; (4) good CT image quality and complete clinical data; and (5) age more than 18 years old. The exclusion criteria were: (1) chronic pancreatitis and recurrent AP; (2) serum lipase or amylase activity elevated by other causes (renal impairment or hepatobiliary, gastroduodenal, intestinal and neoplastic causes, etc.) [19]; (3) pre-existing neoplasms or inflammation in the abdomen and thorax; and (4) pre-existing pleural effusion or pleural effusion caused by other causes (cirrhosis and hypoproteinemia, cancer, congestive heart failure, pneumonia and pulmonary embolism, etc.) [20]. In all, 597 AP patients were initially identified and 132 of them were excluded because of the exclusion criteria. Thus, 465 AP patients were included in this study (Figure 1).

### CT examination

The CT scanners used in the three medical centres include SOMATOM Definition AS (Siemens Healthineers, Germany), SOMATOM Definition Flash (Siemens Healthineers, Germany) and Revolution CT (GE Healthcare, USA). Although different hospitals used different CT equipment, their scanning parameters and scanning methods were almost at the same level. All the patients’ chest CT scan were also performed at the same time as their upper abdominal CT. For the first author’s institution, a Revolution CT scanner was mainly used.

For the non-enhanced chest and upper abdominal CT scanning, each patient was asked to lie in supine position and hold the breath at the end of deep inspiration. The scanning range was from apex of the lung to the upper abdomen at the level of bilateral iliac crest. The main acquisition parameters were as follows: tube voltage = 120 kV; tube current = 300–600 mAs (SmartmA was used); field-of-view (FOV) = (35.0–40.0) cm; collimation = 80 mm; gantry rotation time of = 0.5 s; pitch = 0.992:1; matrix = 512 × 512; slice thickness = 5.0 mm; reconstruction increment = 1.25 mm. For the contrast enhanced upper abdominal CT (CECT) scanning, after a routine non-enhanced scan, arterial and portal-venous phase CECT were performed after 25–30 and 65–70 s delays following the intravenous administration of iodinated contrast material (Iodixanol 320, Jiangsu Hengrui Medicine Co., Ltd, China) at 1.5 mL per kilogram at a rate of 3.0–3.5 mL/s by using a pump injector (Ulrich CT Plus 150, Ulrich Medical, USA). The machine is designed to automatically generate radiation dose data, but at present it was not our intention to explore the relationship between radiation dose and AP.

### CT image analysis

All the thoracic and abdominal CT images were transferred to the picture archiving and communication system (PACS) station (INFINITT PACS, INFINITT Healthcare Co. Ltd., South Korea) for interpretation. The interpretation was independently performed by two radiologists (each with six and 10 years of experience in thoracic and abdominal CT imaging) without knowing the clinical data. If there were any disagreements, a third reviewer (with more than 15 years of experience in thoracic and abdominal CT imaging) was consulted. Whenever possible, the CTSI and EPIC scores on AP were calculated for each individual [21,22]. Based on the CTSI and EPIC scoring systems, the AP patients with less than four points were placed into the mild subgroup while those with four or greater points were placed into the severe subgroup.

### Measurement of PEV

**Figure 2.**
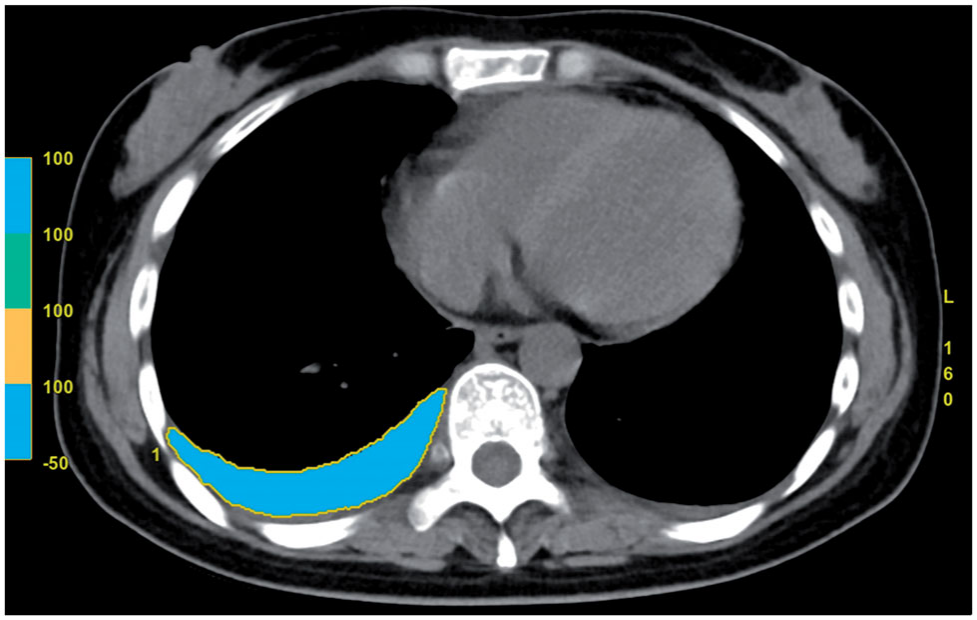
The measurement of pleural effusion volume with chest CT images.

For the measurement of PEV, it was carried on the image post-processing station (AW 4.7, GE Healthcare, USA). The same two radiologists who interpreted the CT images independently performed the PEV measurement without knowing the clinical data. The average value of the two measurements was taken as the final statistical results. First, the pleural fluid and its surrounding margin on each slice of the chest CT images were outlined with the usage of an electronic cursor. Next, the system software was used to measure the CT value of each voxel in the selected region of interest (ROI), after which CT value of all the ROIs with the range of “min −50 to max 100” HU was regarded as pleural effusion. Then, all the ROIs and the total slice thickness were summed with the system software and the total volume of the pleural effusion was finally measured (Figure 2). The total amount of PEV in each patient was the sum of bilateral pleural effusion, and the time taken for each measurement was recorded in seconds. To evaluate the intra-observer and inter-observer variability, 100 AP patients with positive pleural effusion were randomly selected, and the PEV measurements were conducted once again with a four-week interval to avoid potential recall bias.

### Severity of acute pancreatitis and clinical outcomes

All the patients’ electronic medical records were retrospectively reviewed by two gastroenterologists (each with 12 and 15 years of experience in gastroenterology) and the data were collected in consensus. If there were any disagreements, a third reviewer (with more than 20 years of experience in gastroenterology) was consulted. The following clinical outcomes were collected: (1) clinical scoring systems on AP (including the Ranson, BISAP, Marshall, and APACHE II scores); (2) duration of hospitalization (in days); (3) severity of AP according to the 2012 RAC [9–14]; (4) evidence of infection [9,13–15]; (5) need for therapeutic procedures (percutaneous catheter drainage, surgical necrosectomy or both) [10,13,14]; (6) ICU admission; (7) evidence of organ failure [23]; or (8) death. The relevant data regarding fluid therapy administered to the patients were not collected as varied fluid therapy strategies might have been adopted by different gastroenterologists to different AP patients, and secondly, it was not the topic of this study.

The C-reactive protein (CRP) levels were measured 48 h after the onset of AP symptoms [15]. The BISAP, Marshall, and APACHE-II scores were calculated by using data from the first 24 h of admission while the Ranson score was calculated by using data from the first 48 h of admission [23–26]. Based on the CRP levels, the AP patients with <150.0 mg/L were divided into the mild subgroup while those with 150.0 mg/L or greater were divided into the severe subgroup [15]. In the Ranson scoring system, the AP patients with less than three points were divided into the mild subgroup while those with three points or greater were divided into the severe subgroup [24]. In the BISAP scoring system, the AP patients with less than three points were classified as mild while those with three points or greater were divided into the severe subgroup [25]. In the modified Marshall scoring system, two points or greater in one of the cardiovascular, renal and respiratory systems was defined as organ failure [23]. Transient organ failure lasts <48 h while persistent organ failure lasts more than 48 h [23]. In the APACHE II scoring system, the AP patients with less than eight points were placed into the mild subgroup while those with eight points or greater were placed into the severe subgroup [26].

### Statistical analysis

All the descriptive data were expressed as frequency, mean ± standard deviation (*SD*), or median and were compared by using the Chi-squared test or Fisher's exact test, Student *t*-test or Mann–Whitney *U* test, Kruskal–Wallis *H* test, and Student–Newman–Keuls test, as appropriate. The normal distribution of the data was analyzed by visual (probability and histogram graphs) and analytical methods (Shapiro–Wilk or Kolmogorov–Smirnov test).

The inter-observer consistency for the frequency of pleural effusion was investigated by using the kappa (*κ*) statistic, and the intra-class and inter-class correlation coefficients (ICC) analysis were used to evaluate the intra-observer and inter-observer agreements for the measurements of PEV. As previous researchers have reported, a κ statistic of 0.81–1.00, 0.61–0.80, and 0.41–0.60 was considered as excellent, good, and moderate agreement, respectively [15]. ICC values of >0.90, 0.75–0.90, 0.50–0.75, and <0.50 were indicative of excellent, good, moderate, and poor agreement, respectively [27].

The relationship between PEV and duration of hospitalization, CRP levels, Ranson, BISAP, Marshall, APACHE II, CTSI, and EPIC scoring systems were performed by using the Spearman rank correlation coefficient. A Spearman rank correlation coefficient of 0.500–1.00, 0.300–0.490, 0.100–0.290, and 0.090–0.099 were considered to indicate strong, moderate, weak, and no correlation, respectively [28]. The receiver operating characteristic curves (ROC) were constructed to identify the optimal cut-off values of PEV, CRP levels, Ranson, BISAP, Marshall, APACHE II, CTSI, and EPIC scoring systems for predicting severity of AP (both moderately severe and severe AP were included in the severe AP subgroup), infection, procedure, ICU admission, and organ failure (both transient and persistent organ failure were included in the organ failure subgroup). In addition, the area under the ROC curves (AUC) of the parameters were calculated and pairwise comparisons of the AUCs were performed by using the method of Delong et al. [29].

Statistical analyses were performed by using the GraphPad Prism 8.0.0 (https://www.graphpad.com/scientific-software/prism/) and MedCalc 19.5.3 (https://www.medcalc.org/index.php) software, and the results were considered statistically significant if the *p*-values were <.05.

## Results

### Population

A total of 465 patients with AP were enrolled with a mean age of 54.6 (range, 22–85 years old). Of these, 253 (54.4%, 253/465) were male, with a mean age of 55.3 (range, 22–78 years old); 212 (45.6%, 212/465) were female, with a mean age of 53.8 (range, 25–85 years old). There was no significant difference in age between the genders (*p* = .3785). The mean time of AP onset was two days (range, 1–3 days); the mean time of CT examination after AP onset was 2.2 days (range, 2–6 days); and the mean time of interval was 1.8 days (0–5 days).

For the aetiology of AP, gallstone, hypertriglyceridaemia, alcohol abuse, and post-ERCP were observed in 171 (36.8%, 171/465), 122 (26.2%, 122/465), 57 (12.3%, 57/465), and 12 (2.6%, 12/465) patients, respectively. However, there were 16 (3.4%, 171/465) and 87 (18.7%, 87/465) patients with the causes of other (autoimmune and trauma) or unknown (idiopathic) natures. Basic characteristics and aetiology of AP in the 465 patients are shown in Table 1.

**Table 1.** Basic characteristics and aetiology of acute pancreatitis in 465 patients.

Characteristics	Datum
Patient characteristics	
Age (years)^†^	54.6 (22–85)
Male	55.3 (22–78)
Female	53.8 (25–85)
Female/male	0.84 (212/253)
*Time of AP onset (day)^†^	2.0 (1–3)
Time of CT examination after AP onset (day)^†^	2.2 (2–6)
Time of interval (day)^†^	1.8 (0–5)
Cause of AP^Φ^	
Gallstone	171 (36.8%)
Hypertriglyceridaemia	122 (26.2%)
Alcohol abuse	57 (12.3%)
Post-ERCP	12 (2.6%)
Other	16 (3.4%)
Unknown	87 (18.7%)

AP: acute pancreatitis; ERCP: endoscopic retrograde cholangiopancreatography.

^†^Data are means, with ranges in parentheses.

^Φ^Data are numbers of patients, with percentages in parentheses.

*The onset of acute pancreatitis is defined as the time of onset of abdominal pain and the time interval between onset of abdominal pain and CT examination.

### Clinical and severity outcomes of acute pancreatitis

Based on the 2012 RAC, 243 (52.3%, 243/465), 195 (41.9%, 195/465), and 27 (5.8%, 27/465) patients were diagnosed as mild, moderately severe and severe AP, respectively. Meanwhile, 409 (88.0%, 409/465) and 56 (12.0%, 409/465) patients were identified as IEP and necrotizing pancreatitis, respectively. Among the 56 patients with necrotizing pancreatitis, 3 (5.4%, 3/56) were pancreatic only, 14 (25%, 14/56) were peripancreatic only, and 39 (69.6%, 39/56) were both pancreatic and peripancreatic. The median time of hospitalization was 18.3 days (range, 2–85 days). The patients who had suffered from infection, needed therapeutic procedures, ICU admission, organ failure, or death were 116 (24.9%, 116/465), 69 (14.8%, 69/465), 57 (12.3%, 57/465), 85 (18.3%, 85/465), and 19 (4.1%, 19/465), respectively.

For organ failure in 85 patients (18.3%, 85/465), 58 patients (12.5%, 58/465) had transient organ failure (respiratory failure in 35 and renal failure in 23) and 27 patients (5.8%, 27/465) had persistent organ failure (respiratory failure in 15, renal failure in 12). None of the patients had any cardiovascular failure. No transient or persistent organ failure were found in the rest of the 380 patients (81.7%, 380/465).

The mean CRP levels, Ranson, BISAP, Marshall, APACHE II, CTSI, and EPIC scores were 162.8 ± 88.2 mg/L (range, 15–324 mg/L), 3.6 ± 2.9 points (range, 0–9 points), 2.6 ± 1.7 points (range, 0–5 points), 3.2 ± 2.9 points (range, 0–9 points), 7.1 ± 6.5 points (range, 0–45 points), 3.6 ± 3.2 points (range, 0–9 points), 3.8 ± 2.2 points (range, 0–7 points), respectively. Clinical and severity outcomes of AP in the 465 patients are depicted in Table 2.

**Table 2.** Clinical and severity outcomes of acute pancreatitis in 465 patients.

Characteristics	Datum
Clinical outcomes	
Mild AP	243 (52.3%)
Moderately severe AP	195 (41.9%)
Severe AP	27 (5.8%)
Duration of hospitalization (day)*	18.3 (2–85)
Infection	116 (24.9%)
Need for intervention	69 (14.8%)
ICU admission	57 (12.3%)
Organ failure	85 (18.3%)
Death	19 (4.1%)
CRP level (mg/L)	
Mild (<150)	226 (48.6%)
Severe (≥150)	239 (51.4%)
Ranson Score	
Mild (<3)	214 (46.0%)
Severe (≥3)	251 (54.0%)
BISAP Score	
Mild (<3)	226 (48.6%)
Severe (≥3)	239 (51.4%)
Marshall Score	
Mild (<2)	233 (50.1%)
Severe (≥2)	232 (49.9%)
APACHE II Score	
Mild (<8)	234 (50.3%)
Severe (≥8)	231 (49.7%)
CTSI Score	
Mild (<4)	224 (48.2%)
Severe (≥4)	241 (51.8%)
EPIC Score	
Mild (<4)	228 (49.0%)
Severe (≥4)	237 (51.0%)

AP: acute pancreatitis; CRP: C-reactive protein; BISAP: bedside index for severity in acute pancreatitis; APACHE II: acute physiology and chronic health evaluation II; CTSI: computed tomography severity index; EPIC: extrapancreatic inflammation on computed tomography; ICU: Intensive care unit.

Unless otherwise indicated, data are numbers of patients, with percentages in parentheses.

*Data is median, with range in parenthesis.

### Pleural effusion on chest CT

The inter-observer consistency for the frequency of pleural effusion on the chest CT imaging was investigated by using the kappa (*κ*) statistic and a *κ* value of 0.925 (*p* < .0001) showed there was a good agreement between the two radiologists. There were good agreements both in inter-observer and intra-observer tests because of the ICC > 0.75 (95% confidence interval, 0.872–0.973, and 0.961–0.986, respectively) for the measurements of PEV on the chest CT images.

**Figure 3.**
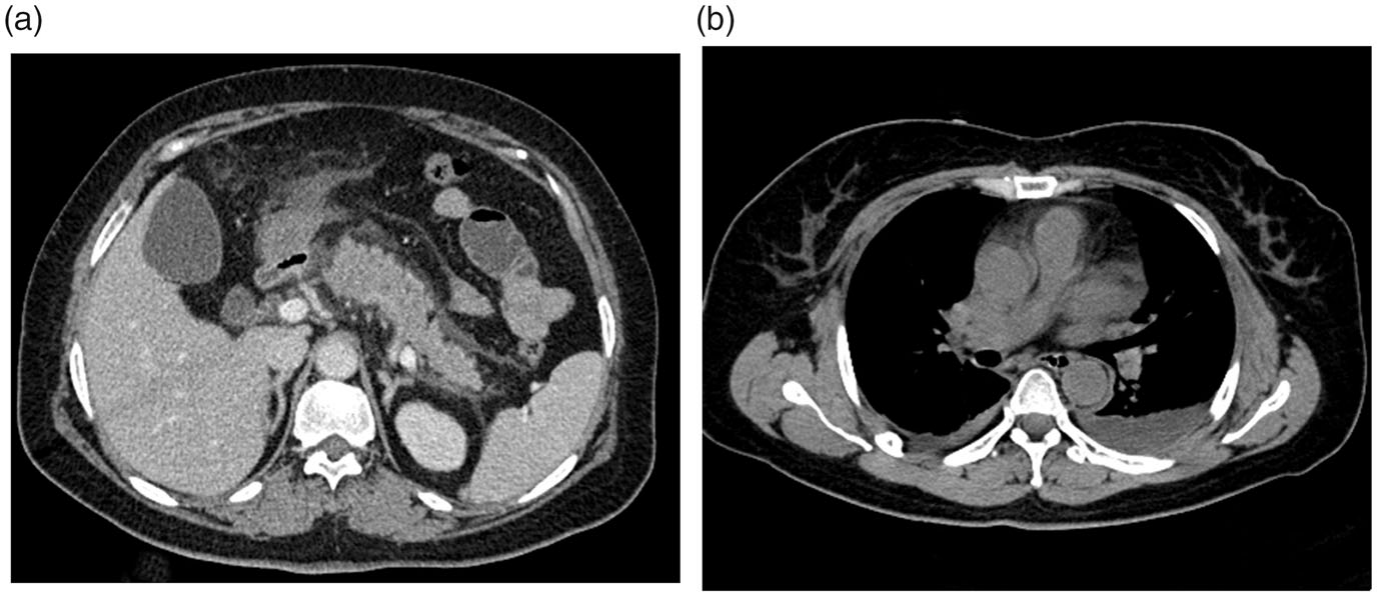
Mild interstitial oedematous pancreatitis (IEP) in a 57-year-old female (CRP level of 28 mg/L, Ranson, BISAP, Marshall, APACHE II, CTSI, and EPIC of 1, 1, 0, 4, 4, and 3 points, respectively; there was no infection, procedure, ICU admission, organ failure, or death on this patient). Abdominal axial contrast-enhanced CT at the portal phase (A) showed there was a small amount of acute peripancreatic fluid collection (APFC) around the pancreas. Axial chest image CT (B) showed there was a small amount of bilateral pleural effusion.

**Figure 4.**
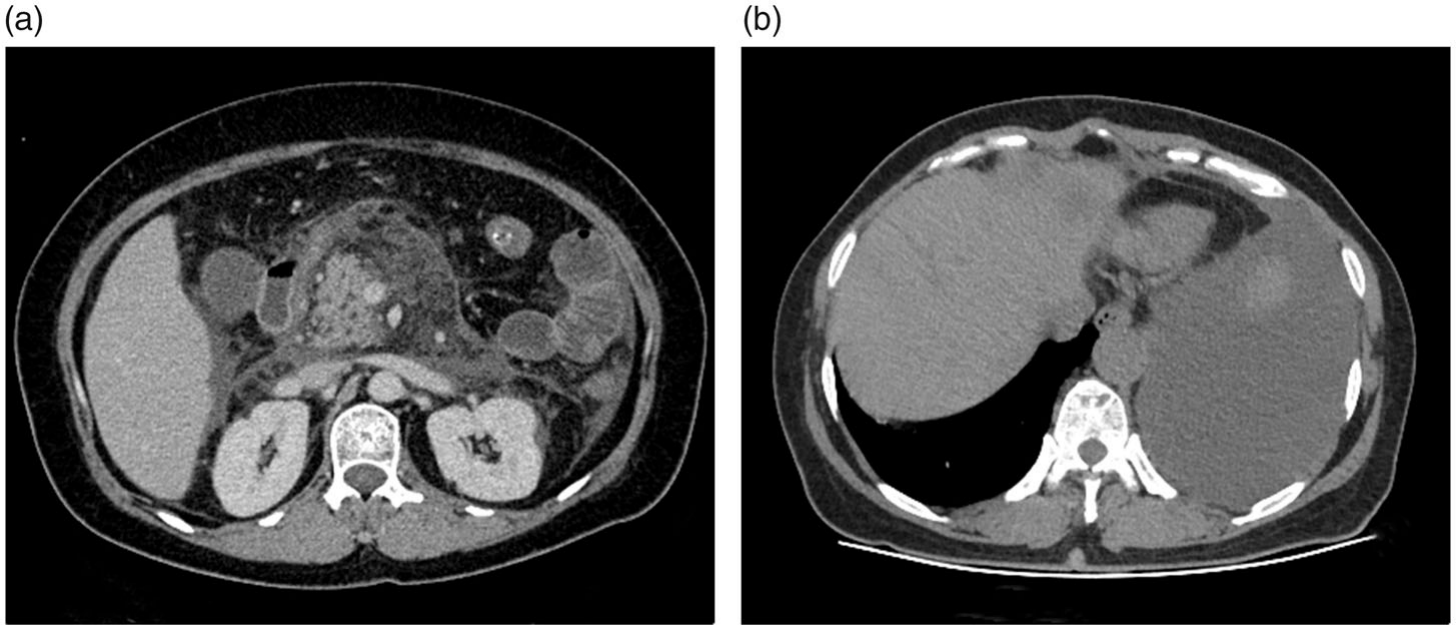
Severe necrotizing pancreatitis (combined pancreatic and peripancreatic) in a 42-year-old male (CRP level of 253 mg/L, Ranson, BISAP, Marshall, APACHE II, CTSI, and EPIC of 5, 4, 18, 6, 6, and 3 points, respectively; positive for infection and organ failure while no procedure was done, no ICU admission, or death on this patient). Abdominal axial contrast-enhanced CT at the portal phase (A) showed there was a large amount of acute necrotic collection (ANC) around the pancreas. Axial chest image CT (B) showed there was a large amount of left pleural effusion.

In the 465 AP patients, 232 (49.9%, 232/465) had positive pleural effusion findings. Out of these, 72.4% (168/232) had bilateral effusion (Figure 3); 23.7% (55/232) had only the left side effusion (Figure 4) while 3.9% (9/232) had right sided effusion. There were significant differences among the three groups regarding the presence of pleural effusion (all the *p*-values were <.0001). The prevalence of pleural effusion in different level of CRP and scoring systems of the 465 AP patients were also observed with significant differences present between the different subgroups (all the *p*-values were <.0001, Table 3).

**Table 3.** Prevalence of pleural effusion in different level of C-reactive protein and scoring systems in 465 patients with acute pancreatitis.

Characteristics	Pleural effusion		*p*-Value
	Present (*n* = 232)	Absent (*n* = 233)	
CRP level (mg/L)			
Mild (<150, *n* = 226)	31 (13.4)	195 (83.7)	<.0001
Severe (≥150, *n* = 239)	201 (86.6)	38 (16.3)	
Ranson Score			
Mild (<3, *n* = 214)	4 (1.7)	210 (90.1)	<.0001
Severe (≥3, *n* = 251)	228 (98.3)	23 (9.9)	
BISAP Score			
Mild (<3, *n* = 226)	9 (3.9)	217 (93.1)	<.0001
Severe (≥3, *n* = 239)	223 (96.1)	16 (6.9)	
Marshall Score			
Mild (<2, *n* = 233)	23 (9.9)	210 (90.1)	<.0001
Severe (≥2, *n* = 232)	209 (90.1)	23 (9.9)	
APACHE II Score			
Mild (<8, *n* = 234)	5 (2.2)	229 (98.3)	<.0001
Severe (≥8, *n* = 231)	227 (97.8)	4 (1.7)	
CTSI Score			
Mild (<4, *n* = 224)	20 (8.6)	204 (87.6)	<.0001
Severe (≥4, *n* = 241)	212 (91.4)	29 (12.4)	
EPIC Score			
Mild (<4, *n* = 228)	28 (12.1)	200 (85.8)	<.0001
Severe (≥4, *n* = 237)	204 (87.9)	33 (14.2)	

CRP: C-reactive protein; BISAP: bedside index for severity in acute pancreatitis; APACHE II: acute physiology and chronic health evaluation II; CTSI: computed tomography severity index; EPIC: extrapancreatic inflammation on computed tomography.

Data are numbers of patients, with percentages in parentheses.

The mean PEV was 98.8 ± 113.2 mL (range, 0.0–1328.0 mL) and the mean time we spent on measuring the PEV on the CT images was <3 min (range, 1–5 min).

### The correlations between PEV and duration of hospitalization, CRP levels and different scoring systems

**Figure 5.**
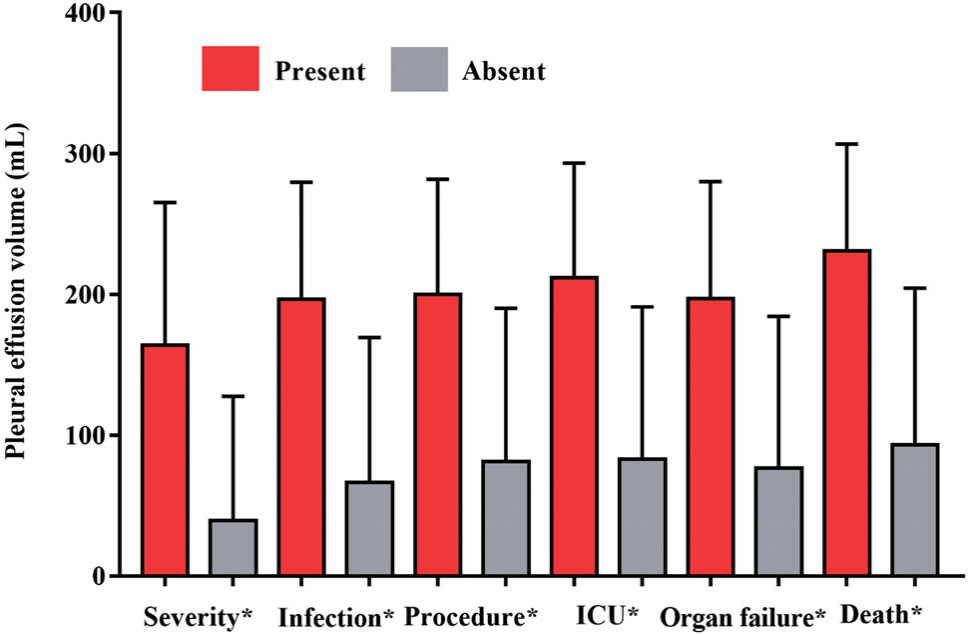
Bar graph shows mean pleural effusion volume in millilitres (error bars = 95% CIs) for each clinical outcome. *p < .0001.

The Spearman correlation coefficient between the PEV and the duration of hospitalization was 0.5273 (95% CI = 0.4868–0.5815, *p <* .0001), which indicating a strong and significant correlation between the two parameters. There was also a strong and significant correlation between the PEV and the CRP levels (*r* = 0.5717, 95% CI = 0.5051–0.6315, *p* < .0001). Besides that, we found a strong and significant correlation between the PEV and the Ranson, BISAP, Marshall, APACHE II, CTSI, and EPIC scores (all *r* > 0.05 and all the *p*-values are <.0001). In addition, there was also a statistically significant correlation between the PEV and the occurrence of serve AP, infection, therapeutic procedures, ICU admission, organ failure or death (Figure 5, all the *p*-values are <.0001). The correlations between PEV and duration of hospitalization, CRP levels and different scoring systems are demonstrated in the Table 4.

**Table 4.** The relationship between pleural effusion volume and C-reactive protein level, and different scoring systems in 465 patients with acute pancreatitis.

Characteristics	Pleural effusion volume		
	*r*	95% CI	*p*-Value
Duration of hospitalization (day)	0.5273	0.4868–0.5825	<.0001
CRP level (mg/L)	0.5717	0.5051–0.6315	<.0001
Ranson Score	0.7085	0.6585–0.7522	<.0001
BISAP Score	0.7149	0.6659–0.7578	<.0001
Marshall Score	0.6098	0.5475–0.6654	<.0001
APACHE II Score	0.7598	0.7172–0.7968	<.0001
CTSI Score	0.6249	0.5643–0.6788	<.0001
EPIC Score	0.6102	0.5479–0.6658	<.0001

CRP: C-reactive protein; BISAP: bedside index for severity in acute pancreatitis; APACHE II: acute physiology and chronic health evaluation II; CTSI: computed tomography severity index; EPIC: extrapancreatic inflammation on computed tomography; 95% CI: 95% confidence interval.

### PEV, CRP levels, and different scoring systems for predicting severity and clinical outcomes of acute pancreatitis

On the basis of PEV, the ROC curve yielded an AUC of 0.8158 (95% CI = 0.7747–0.8568, *p* < .0001) for predicting severe AP, with a threshold of 69.00 mL, and the sensitivity (%) and specificity (%) were 84.23 (95%CI, 78.76–88.77) and 81.07 (95% CI, 75.57–85.79), respectively.

For predicting infection, PEV was observed an AUC of 0.8311 (95% CI: 0.7956–0.8665, *p* < .0001), with a threshold of 65.00 mL, and the sensitivity (%) and specificity (%) were 85.69 (95% CI: 80.23–88.59) and 77.34 (95% CI: 72.14–82.23), respectively.

In predicting therapeutic procedures, PEV was noted an AUC of 0.7987 (95% CI: 0.7569–0.8406, *p* < .0001), with a threshold of 71.00 mL, and the sensitivity (%) and specificity (%) were 87.10 (95% CI: 79.92–89.65) and 69.34 (95% CI: 64.32–74.22), respectively.

As to predict ICU admission, PEV was found an AUC of 0.8148 (95% CI: 0.7714–0.8582, *p* < .0001), with a threshold of 75.00 mL, and the sensitivity (%) and specificity (%) were 88.25 (95% CI: 80.61–89.96) and 67.84 (95% CI: 62.89–72.69), respectively.

As for predicting organ failure, PEV provided an AUC of 0.8045 (95% CI: 0.7650–0.8440, *p* < .0001), with a threshold of 78.00 mL, and the sensitivity (%) and specificity (%) were 87.65 (95% CI: 81.76–89.71) and 71.84 (95% CI: 66.75–76.75), respectively.

For predicting death, PEV provided an AUC of 0.8253 (95% CI: 0.7609–0.8897, *p* < .0001), with a threshold of 82.00 mL, and the sensitivity (%) and specificity (%) were 64.74 (95% CI: 53.97–79.87) and 70.73 (95% CI: 65.22–78.38), respectively.

PEV, CRP levels and different scoring systems for predicting the severity and clinical outcomes of AP with AUC, optimal threshold points, sensitivity and specificity are presented in the Table 5 (p-values were <.0001).

**Table 5.** Receiver operating characteristic curve analysis of pleural effusion volume, C-reactive protein and different scoring systems for predicating severity and clinical outcomes of acute pancreatitis in 465 patients.

Characteristics	AUC (95% CI)	Threshold	Sensitivity (%, 95% CI)	Specificity (%, 95% CI)	*p*-Value
Severe AP					
PEV (mL)	0.8158 (0.7747–0.8568)	≥69	84.23 (78.76–88.77)	81.07 (75.57–85.79)	<.0001
CRP level (mg/L)	0.7003 (0.6526–0.7481)	≥208	69.37 (62.85–75.36)	68.31 (62.06–74.11)	<.0001
Ranson Score	0.8479 (0.8098–0.886)	≥3	91.44 (86.96–94.77)	80.25 (74.68–85.06)	<.0001
BISAP Score	0.7693 (0.7252–0.8135)	≥3	80.18 (74.32–85.21)	74.90 (68.96–80.22)	<.0001
Marshall Score	0.7264 (0.6794–0.7734)	≥2	73.87 (67.57–79.52)	72.02 (65.92–77.57)	<.0001
APACHE II Score	0.8676 (0.8315–0.9036)	≥10	75.68 (69.48–81.17)	92.18 (88.06–95.23)	<.0001
CTSI Score	0.7203 (0.6732–0.7675)	≥4	75.23 (69.01–80.76)	69.55 (63.34–75.27)	<.0001
EPIC Score	0.7098 (0.6623–0.7572)	≥4	71.62 (65.2–77.45)	67.90 (61.63–73.73)	<.0001
Infection					
PEV (mL)	0.8311 (0.7956–0.8665)	≥65	85.69 (80.23 − 88.59)	77.34 (72.14–82.23)	<.0001
CRP level (mg/L)	0.7330 (0.6853–0.7807)	≥176	83.62 (75.62–89.84)	62.18 (56.86–67.29)	<.0001
Ranson Score	0.7804 (0.7395–0.8213)	≥3	79.31 (70.80–86.27)	65.62 (60.37–70.59)	<.0001
BISAP Score	0.7935 (0.7510–0.8360)	≥3	84.83 (79.08–88.08)	73.04 (67.74–78.12)	<.0001
Marshall Score	0.7670 (0.7189–0.8151)	≥2	79.31 (70.80–86.27)	69.05 (63.91–73.87)	<.0001
APACHE II Score	0.8656 (0.8321–0.8991)	≥10	87.93 (80.58–93.24)	75.64 (70.79–80.06)	<.0001
CTSI Score	0.7524 (0.7039–0.8010)	≥4	75.00 (66.11–82.57)	76.76 (71.55–81.69)	<.0001
EPIC Score	0.7526 (0.7048–0.8003)	≥4	87.07 (79.57–92.58)	71.03 (65.70–76.18)	<.0001
Procedure					
PEV (mL)	0.7987 (0.7569–0.8406)	≥71	87.10 (79.92–89.65)	69.34 (64.32–74.22)	<.0001
CRP level (mg/L)	0.7314 (0.6779–0.7848)	≥182	88.41 (78.43–94.86)	67.58 (62.54–72.50)	<.0001
Ranson Score	0.7712 (0.7277–0.8148)	≥3	84.06 (73.26–91.76)	61.11 (56.11–65.94)	<.0001
BISAP Score	0.7692 (0.7245–0.8139)	≥3	67.97 (55.48–79.76)	69.44 (64.65–73.95)	<.0001
Marshall Score	0.7650 (0.7126–0.8174)	≥2	79.71 (68.31–88.44)	63.38 (58.43–68.14)	<.0001
APACHE II Score	0.8197 (0.7818–0.8576)	≥10	89.86 (80.21–95.82)	68.43(63.61–72.99)	<.0001
CTSI Score	0.7331 (0.6786–0.7876)	≥4	76.81(65.09–86.13)	62.12 (57.14–66.92)	<.0001
EPIC Score	0.7263 (0.6722–0.7805)	≥4	69.57 (57.31–80.08)	65.66 (60.75–70.33)	<.0001
ICU admission					
PEV (mL)	0.8148 (0.7714–0.8582)	≥75	88.25 (80.61–89.96)	67.84 (62.89–72.69)	<.0001
CRP level (mg/L)	0.7274 (0.6727–0.7821)	≥178	79.47 (68.48–86.04)	65.88 (60.91–70.76)	<.0001
Ranson Score	0.7518 (0.7054–0.7983)	≥3	82.46 (70.09–91.25)	69.56 (64.62–74.36)	<.0001
BISAP Score	0.7692 (0.7211–0.8174)	≥3	67.89 (54.08–80.86)	68.63 (63.88–73.10)	<.0001
Marshall Score	0.7505 (0.6962–0.8048)	≥2	78.95 (66.11–88.62)	72.01 (67.10–76.74)	<.0001
APACHE II Score	0.8147 (0.7748–0.8546)	≥10	80.70 (68.09–89.95)	70.59 (65.91–74.97)	<.0001
CTSI Score	0.7299 (0.6277–0.7876)	≥4	75.44 (62.24–85.87)	70.78 (65.86–75.55)	<.0001
EPIC Score	0.7309 (0.6760–0.7858)	≥4	70.18 (56.60–81.57)	64.71 (59.85–69.34)	<.0001
Organ failure					
PEV (mL)	0.8045 (0.7650–0.8440)	≥78	87.65 (81.76–89.71)	71.84 (66.75–76.75)	<.0001
CRP level (mg/L)	0.7417 (0.6928–0.7906)	≥185	89.41 (80.85–95.04)	69.74 (64.61–74.71)	<.0001
Ranson Score	0.7660 (0.7240–0.8081)	≥3	81.18 (71.24–88.84)	62.37 (57.28–67.26)	<.0001
BISAP Score	0.7901 (0.7487–0.8315)	≥3	88.82 (83.62–89.97)	69.21 (64.08–74.19)	<.0001
Marshall Score	0.7836 (0.7352–0.832)	≥2	82.35 (72.57–89.77)	65.79 (60.78–70.55)	<.0001
APACHE II Score	0.8410 (0.8060–0.8760)	≥10	88.24 (79.43–94.21)	70.53 (65.66–75.07)	<.0001
CTSI Score	0.7437 (0.6923–0.7952)	≥4	75.29 (64.75–84.01)	63.42 (58.36–68.27)	<.0001
EPIC Score	0.7526 (0.7019–0.8033)	≥4	71.76 (60.96–81.00)	67.63 (62.67–72.31)	<.0001
Death					
PEV (mL)	0.8253 (0.7609–0.8897)	≥82	64.74 (53.97–79.87)	70.73 (65.22–78.38)	<.0001
CRP level (mg/L)	0.8234 (0.7706–0.8762)	≥216.5	73.68 (68.43–82.16)	67.35 (63.18–75.16)	<.0001
Ranson Score	0.7223 (0.6511–0.7936)	≥5	79.68 (67.86–82.85)	83.95 (69.25–89.37)	.0010
BISAP Score	0.7122 (0.6357–0.7887)	≥3	68.89 (63.65–79.92)	76.82 (62.28–82.35)	.0017
Marshall Score	0.8081 (0.7350–0.8811)	≥4	65.95 (54.43–73.95)	62.47 (55.85–69.88)	<.0001
APACHE II Score	0.7821 (0.7252–0.8389)	≥11	84.21 (70.42–96.62)	73.96 (65.47–78.68)	<.0001
CTSI Score	0.7389 (0.6592–0.8186)	≥5	78.95 (64.43–83.95)	67.85 (63.11–72.48)	.0004
EPIC Score	0.6895 (0.6297–0.7432)	≥5	65.63 (56.96–71.55)	68.99 (62.29–75.54)	.0121

AP: acute pancreatitis; PEV: pleural effusion volume; CRP: C-reactive protein; BISAP: bedside index for severity in acute pancreatitis; APACHE II: acute physiology and chronic health evaluation II; CTSI: computed tomography severity index; EPIC: extrapancreatic inflammation on computed tomography; ICU: intensive care unit; AUC: area under the receiver operating characteristic curve; 95% CI: 95% confidence interval.

### Comparison of PEV, CRP levels, and different scoring systems for predicting severity and clinical outcomes of acute pancreatitis

**Figure 6.**
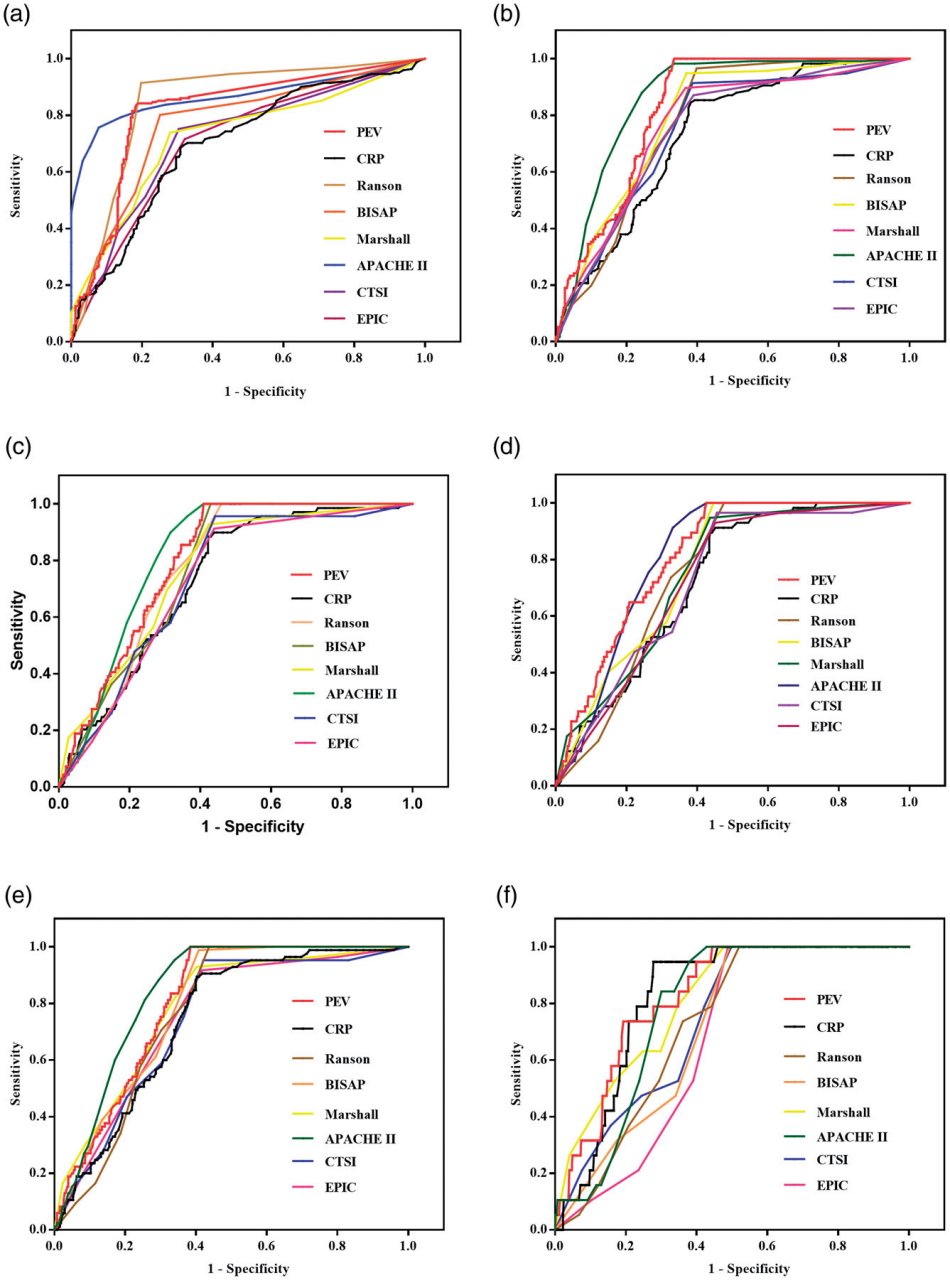
Receiver operating characteristic curves (ROC) of the pleural effusion volume, C-reactive protein levels and different clinical scoring systems for predicting severe acute pancreatitis (A), infection (B), procedure (C), ICU admission (D), organ failure (E), and death (F).

For predicting severe AP, the accuracy of PEV was significantly higher than that of CRP levels (*p* < .0001), BISAP scores (*p* = .0052), Marshall scores (*p* < .0001), CTSI scores (*p* < .0001), and EPIC scores (*p* < .0001), and its accuracy was similar to that of the Ranson scores (*p* = .0648). However, the accuracy of PEV was significantly lower than that of the APACHE II scores (*p* = .0019). The APACHE II scores demonstrated the highest while the CRP levels demonstrated the lowest AUC of all the parameters for predicting severe AP (*p*-values are <.05, Figure 6(A)).

For predicting infection, the accuracy of PEV was significantly higher than that of CRP levels (*p* = .0002), Ranson scores (*p* = .0222), Marshall scores (*p* = .0179), CTSI scores (*p* = .0036), and EPIC scores (*p* = .0022), and its accuracy was similar to that of BISAP scores (*p* = .1057) and APACHE II scores (*p* = .0955). The CRP levels showed the lowest AUC of all the parameters for predicting infection (*p*-values are <.05, Figure 6(B)).

For predicting therapeutic procedures, the accuracy of PEV was significantly higher than that of CRP levels (*p* = .0267), CTSI scores (*p* = .0285), and EPIC scores (*p* = .0133), and its accuracy was similar to that of Ranson scores (*p* = .3209), BIASP scores (*p* = .2395), Marshall scores (*p* = .2750), and APACHE II scores (*p* = .4015). The CRP levels and EPIC scores showed a similar AUC for predicting therapeutic procedures (*p* = .8396) and both were found with the lowest AUC of all the parameters for predicting procedure (*p*-values are <.05, Figure 6(C)).

For predicting ICU admission, the accuracy of PEV was significantly higher than that of CRP levels (*p* = .0060), Ranson scores (*p* = .0419), CTSI scores (*p* = .0084), and EPIC scores (*p* = .0067). Its accuracy was similar to than that of BISAP scores (*p* = .1105), Marshall scores (*p* = .0505), and APACHE II scores (*p* = .9970). The CRP levels was noted with the lowest AUC of all the parameters for predicting ICU admission (*p*-values are <.05, Figure 6(D)).

For predicting organ failure, the accuracy of PEV was significantly higher than that of CRP levels (*p* = .0247), CTSI scores (*p* = .0325), and EPIC scores (*p* = .0582). Its accuracy was similar to that of Ranson scores (*p* = .1180), BIASP scores (*p* = .5244), Marshall scores (*p* = .4550), and APACHE II scores (*p* = .1044). The CRP levels was identified with the lowest AUC of all the parameters for predicting organ failure (*p*-values are <.05, Figure 6(E)).

For predicting death, the accuracy of PEV was significantly higher than that of Ranson scores (*p* = .0223), BIASP scores (*p* = .0274), CTSI scores (*p* = .0331), and EPIC scores (*p* = .0405). Its accuracy was similar to that of CRP levels (*p* = .1736), Marshall scores (*p* = .3276), and APACHE II scores (*p* = .2518). The EPIC scores were identified with the lowest AUC of all the parameters for predicting death (*p*-values are <.05, Figure 6(F)).

Figure 6 and Table 6 shows the comparison of PEV, CRP levels and different scoring systems for predicting severity and clinical outcomes of AP.

**Table 6.** Pairwise comparison of AUC of pleural effusion volume, C-reactive protein and different scoring systems in 465 patients with acute pancreatitis.

Characteristics	Severe AP		Infection		Procedure		ICU admission		Organ failure	
	*Z* value	*p*-Value	*Z* value	*p*-Value	*Z* value	*p*-Value	*Z* value	*p-*Value	*Z* value	*p*-Value
PEV *vs.* CRP level	5.427	<.0001*	3.698	.0002*	2.216	.0267*	2.750	.0060*	2.247	.0247*
PEV *vs.* Ranson Score	1.847	.0648	2.287	.0222*	0.993	.3209	2.034	.0419*	1.563	.1180
PEV *vs.* BISAP Score	2.792	.0052*	1.618	.1057	1.176	.2395	1.596	.1105	0.637	.5244
PEV *vs.* Marshall Score	4.298	<.0001*	2.367	.0179*	1.092	.2750	1.956	.0505	0.747	.4550
PEV *vs.* APACHE II Score	3.103	.0019*	1.667	.0955	0.839	.4015	0.00377	.9970	1.624	.1044
PEV *vs.* CTSI Score	4.862	<.0001*	2.914	.0036*	2.190	.0285*	2.636	.0084*	2.139	.0325*
PEV *vs.* EPIC Score	5.215	<.0001*	3.058	.0022*	2.477	.0133*	2.712	.0067*	1.894	.0582*
CRP level *vs.* Ranson Score	6.418	<.0001*	1.880	.0601	1.371	.1705	0.793	.4277	0.909	.3634
CRP level *vs.* BISAP Score	3.247	.0012*	2.416	.0157*	1.184	.2363	1.204	.2284	1.730	.0836
CRP level *vs.* Marshall Score	1.341	.1799	1.512	.1305	1.234	.2170	0.732	.4639	1.712	.0869
CRP level *vs.* APACHE II Score	7.558	<.0001*	5.689	<.0001*	3.159	.0016*	2.959	.0031*	3.958	.0001*
CRP level *vs.* CTSI Score	0.993	.3205	0.821	.4119	0.0577	.9540	0.0795	.9367	0.0778	.9380
CRP level *vs.* EPIC Score	0.539	.5897	1.001	.3168	0.202	.8396	0.126	.8998	0.492	.6228
Ranson *vs.* BISAP Score	3.939	.0001*	0.589	.5562	0.0748	.9404	0.563	.5738	0.969	.3324
Ranson *vs.* Marshall Score	5.218	<.0001*	0.511	.6092	0.204	.8385	0.0408	.9675	0.615	.5384
Ranson *vs.* APACHE II Score	0.924	.3553	3.991	<.0001*	2.100	.0357*	2.466	.0137*	3.569	.0004*
Ranson *vs.* CTSI Score	5.971	<.0001*	1.144	.2527	1.265	.2059	0.657	.5110	0.809	.4187
Ranson *vs.* EPIC Score	6.043	<.0001*	1.083	.2789	1.509	.1314	0.673	.5009	0.472	.6370
BISAP *vs.* Marshall Score	2.354	.0186*	1.234	.2172	0.140	.8885	0.587	.5569	0.250	.8024
BISAP *vs.* APACHE II Score	5.785	<.0001*	3.498	<.0005*	2.023	.0431*	1.632	.1027	2.293	.0218
BISAP *vs.* CTSI Score	2.734	.0063*	1.868	.0617	1.209	.2267	1.180	.2382	1.735	.0828
BISAP *vs.* EPIC Score	2.816	.0049*	1.669	.0951	1.372	.1701	1.151	.2497	1.352	.1763
Marshall *vs.* APACHE II Score	6.886	<.0001*	4.242	<.0001*	1.908	.0564	2.066	.0388*	2.216	.0267*
Marshall *vs.* CTSI Score	0.354	.7234	0.680	.4965	1.076	.2821	0.647	.5176	1.533	.1254
Marshall *vs.* EPIC Score	0.887	.3749	0.642	.5207	1.318	.1876	0.574	.5658	1.213	.2251
APACHE II *vs.* CTSI Score	7.282	<.0001*	4.786	<.0001*	2.901	.0037*	2.590	.0096*	3.665	.0002*
APACHE II *vs.* EPIC Score	7.123	<.0001*	4.652	<.0001*	3.316	.0009*	2.782	.0054*	3.411	.0006*
CTSI *vs.* EPIC Score	0.569	.5691	0.00661	.9947	0.239	.8110	0.0333	.9734	0.349	.7269

PEV: pleural effusion volume; AP: acute pancreatitis; ICU: intensive care unit; CRP: C-reactive protein; BISAP: bedside index for severity in acute pancreatitis; APACHE II: acute physiology and chronic health evaluation II; CTSI: computed tomography severity index; EPIC: extrapancreatic inflammation on computed tomography; AUC: area under the receiver operating characteristic curve.

*p < .05.

### PEV for evaluating the severity of organ failure and presence of pancreatic necrosis

The severity of organ failure included persistent organ failure, transient organ failure and no organ failure. The PEV in patients with persistent organ failure was similar to that of transient organ failure (165.2 ± 90.3 *vs.* 136.2 ± 62.5 mL, *p* = .1603), while PEV in patients without organ failure was lower than that of the patients with organ failure (54.9 ± 24.6 *vs.* 150.7 ± 79.0 mL, *p* < .0001). In addition, PEV in patients without pancreatic necrosis was lower than that of the patients with pancreatic necrosis (91.3 ± 52.5 *vs.* 218.6 ± 118.1 mL, *p* < .0001). However, we didn’t investigate the correlations between PEV and extent of pancreatic necrosis because there are three subtypes of necrotizing pancreatitis and each of these subtypes had a small sample.

## Discussion

In a previous study by Meyrignac et al. in 2015 [15], extra pancreatic necrosis volume (EPNV) was found to be significantly associated with the length of the hospital stay, infection, need for surgery or percutaneous intervention, occurrence of organ failure, and death in patients with AP (*p* < .001 for all); and a cut-off of 100.00 mL of EPNV provided more notable information than that of the current Balthazar score, CTSI and CRP level for predicting the occurrence of infection and organ failure (*p* < .001 for all). Afterwards in 2019, in a similar study, Çakar et al. compared EPNV to scoring systems of Balthazar, CTSI, and modified CTSI that was based on CT imaging or laboratory examination of CRP level at 48–72 h, and observed EPNV had a better performance for predicting the occurrence of infection and organ failure in AP patients (*p* = .0001 for all) [17]. Recently in 2020, a study by Pamies-Guilabert et al. reported that pancreatic necrosis volume (PNV) was a new radiological biomarker of AP severity [18]. In their study, with a threshold value of 75.00 mL, PNV was found to be a parameter that conveyed the most accuracy in predicting infection, need for therapeutic procedures, ICU admission and organ failure complications than that of CRP level at 48 h, BISAP, Balthazar and CTSI scoring systems (*p*-values were <.05). However, there are little data on the associations between PEV and severity or clinical outcomes of AP.

This present study evaluated the value of PEV, CRP levels, Ranson scores, BISAP scores, Marshall scores, APACHE II scores, CTSI scores, and EPIC scores for the prediction of severity and clinical outcomes of patients with AP. Our findings include: (1) there were strong and significant correlations between PEV and CRP levels, duration of hospitalization, and the Ranson, BISAP, Marshall, APACHE II, CTSI, and EPIC scores (all *r* > 0.5 and all *p*-values were <.05); (2) for predicting severity, the accuracy of PEV was significantly higher than that of CRP levels, BISAP scores, Marshall scores, CTSI scores, and EPIC scores (*p* < .05 for all), and its accuracy was similar to that of the Ranson scores (*p* > .05) while lower than that of the APACHE II scores (*p* < .05); (3) for predicting infection, the accuracy of PEV was significantly higher than that of CRP levels, Ranson scores, Marshall scores, CTSI scores, and EPIC scores (*p* < .05 for all), and its accuracy was similar to that of BISAP scores and APACHE II scores (*p* > .05 for both); (4) for predicting therapeutic procedures, the accuracy of PEV was significantly higher than that of CRP levels, CTSI scores, and EPIC scores (*p* < .05 for all), and its accuracy was similar to that of Ranson scores, BIASP scores, Marshall scores, and APACHE II scores (*p >* .05 for all); (5) for predicting ICU admission, the accuracy of PEV was significantly higher than that of CRP levels, Ranson scores, CTSI scores, and EPIC scores (*p* < .05 for all), and its accuracy was similar to than that of BISAP scores, Marshall scores, and APACHE II scores (*p >* .05 for all); (6) for predicting organ failure, the accuracy of PEV was significantly higher than that of CRP levels, CTSI scores, and EPIC scores (*p* < .05 for all), and its accuracy was similar to that of Ranson scores, BIASP scores, Marshall scores, and APACHE II scores (*p >* .05 for all); and (7) for predicting death, the accuracy of PEV was significantly higher than that of Ranson, BIASP, CTSI, and EPIC scores (*p* < .05 for all), and its accuracy was similar to that of CRP levels, Marshall scores, and APACHE II scores (*p* > .05 for all).

Peng et al. also studied the value of PEV measured on chest CT in the early prediction of severity and clinical outcomes of AP [28]. In their report, there was a strong association between the PEV and BISAP score (*r* = 0.618, *p* = .000) as well as the CTSI score (*r* = 0.574, *p* = .000), while there was a weak association with the duration of hospitalization (*r* = 0.249, *p* = .000) and the APACHE II score (*r* = 0.298, *p* = .000). In addition, they also described that the PEV hold a similar accuracy (AUC = 0.839) for predicting severe AP to that of the BISAP score (AUC = 0.833), APACHE II score (AUC = 0.860), and CTSI score (AUC = 0.842) (*p*-values >.05 for all). Also, the accuracy of PEV (AUC = 0.783) was similar to that of BISAP score (AUC = 0.784), APACHE II score (AUC = 0.853), and CTSI score (AUC = 0.754) for predicting organ failure (*p*-values >.05 for all). However, there were some drawbacks in the study by Peng et al. [28]. First, as the study was performed in a single centre, it is not sure whether these results can be generalized or not. On the contrary, this present study was a multi-institutional one accomplished in three AP centres. Second, only three clinical outcomes (i.e. duration of hospitalization, severity of AP, and organ failure) and three scoring systems (i.e. BISAP score, APACHE II score, and CTSI score) were included for evaluating. By contrast, this present study also included clinical outcomes of infection, the need for therapeutic procedures, ICU admission, and scoring systems of Ranson score, Marshall score, EPIC score, and CRP levels for investigating.

In our study, we did not find any weak association between the PEV and the duration of hospitalization or the APACHE II score (*r* = 0.249 and 0.298, respectively, and *p* = .000 for both in the study by Peng et al. [28]. On the contrary, in our study, there was a strong association between the PEV and the duration of hospitalization or the APACHE II score (*r* = 0.5273 and 0.7598, respectively, and *p* < .0001 for both in this study). We also did not find the PEV shared a similar accuracy for predicting severe AP to that of the BISAP score, APACHE II score, and CTSI score (AUC = 0.839, 0.833, 0.860, and 0.842, respectively, and *p*-values >.05 for all in the study by Peng et al. [28]. By contrast, for predicting severity of AP, the accuracy of PEV was significantly higher than that of BISAP score and CTSI score (*p* < .05 for both), and its accuracy was significantly lower than that of the APACHE II score (*p* < .05) in our study. There were inconsistencies between the two studies in predicting organ failure of AP either. In our study, the accuracy of PEV was significantly higher than that of CTSI score (*p* < .05), and its accuracy was similar to that of BIASP score and APACHE II score (*p >* .05 for both). While in the study by Peng et al. [28], they stated that the PEV carried a similar accuracy for predicting organ failure in AP to that of the BISAP score, APACHE II score, and CTSI score (AUC = 0.783, 0.784, 0.853, and 0.754, respectively, and *p*-values >.05 for all).

All of those above mentioned discrepancy in the results between the two studies may be mainly due to different number of AP patients included (309 *vs.* 465), while the patients’ clinical conditions also varied largely between these two studies too. In the study by Peng et al. [28], the mean PEV was 41.7 ± 38.0 mL; mean BISAP score was 1.3 ± 1.0 points (range, 0.0–5.0 points), mean APACHE II score was 5.8 ± 5.1 points (range, 0.0–33.0 points), and mean CTSI was 3.7 ± 1.8 points (range, 0.0–10.0 points); 5.5% of patients developed severe AP, and 13.9% of patients developed organ failure. By contrast, in our cohorts, we identified a mean PEV of 98.8 mL; mean BISAP score, APACHE II score, and CTSI score of 2.6 ± 1.7 points (range, 0.0–5.0 points), 7.1 ± 6.5 points (range, 0.0–45.0 points), and 3.6 ± 3.2 points (range, 0.0–9.0 points), respectively; 5.8% of patients developed severe AP, and 18.3% of patients developed organ failure.

In this present study, we also included the CRP levels, Ranson, Marshall, and EPIC score. However, since we mainly focussed on evaluating the value of PEV in the prediction of severity and clinical outcomes of patients with AP in this present study, we do not intend to comprehensively discuss the value of CRP levels, Ranson, BISAP, Marshall, APACHE II, CTSI, and EPIC scores in predicting severity and clinical outcomes of AP. In addition, there have been many studies in the literature that have assessed the above-mentioned parameters in the prediction of severity and clinical outcomes of AP [30–36]. For example, Papachristou et al. [30] compared BISAP score with the Ranson, APACHE-II, and CTSI scoring systems in the prediction of severity, pancreatic necrosis, and death in a prospective cohort of patients with AP. Their results indicated that the BISAP score system is an accurate method for risk stratification in patients with AP while the prognostic accuracy of BISAP score is similar to those of the Ranson, APACHE-II, and CTSI scoring systems. In another study by Gezer et al. [36], they stated that with a cut-off of BISAP score ≥2.0, the BISAP score hold the highest value for severity of AP; whereas with a cut-off of >11.91, the neutrophil-lymphocyte ratio (NLR) shared the highest value for mortality of AP. Of all the radiological scoring systems (the Balthazar, modified CTSI, and EPIC score were included in that study), and with a cut-off value ≥6.0, the EPIC score had the highest AUC (0.773, 95% CI: 0.645–0.900) for severity and 0.851 (95% CI: 0.718–0.983) for mortality. Nevertheless, what we want to highlight here is that since some studies may share the same idea while the others may contradict it, controversies do exist about which of the above clinical, laboratorial, and radiological scoring systems can be best used in the clinical practice and scientific research [30–36]. And that’s maybe why only the modified Marshall scoring system is endorsed in the revised Atlanta classification as a method for determining organ failure [9].

Pleural effusion is one of the most common thoracic complications in AP patients [37]. In previous reports, the prevalence of pleural effusion in patients with AP was shown to be at 3.0–50.0% [37,38]. However, recent studies demonstrated that the prevalence was up to 46.0–72.3% [39,40]. Furthermore, previous investigation also pointed out that pleural effusion in AP patients are usually in the left sides [37]. Nevertheless, in this present study, 49.9% (232/465) of AP had pleural effusion, of which, 72.4% (168/232) were bilateral, 23.7% (55/232) were on the left side, and 3.9% (9/232) on the right sides (*p*-values are <.0001). Some of the results in our study are consistent with those of some previous results [28,37–40]. For example, in the study by Peng et al. [28], the incidence of pleural effusion in patients with AP was reported to be 39.8% (123/309), among which, 65.0% (80/123) were noted in the bilateral sides, 30.1% (37/123) were left sides, and 4.9% (6/123) were in right sides (*p*-values are <.0001). Peng [28] and our study both found that the PEV in patients with persistent organ failure was similar to that of transient organ failure, while the PEV in the patients without organ failure was lower than that of the patients with organ failure (*p* < .05 for both). We also found that the PEV in the patients without pancreatic necrosis was lower than that of the patients with pancreatic necrosis (*p* < .0001) while Peng [28] did not report that point.

There are also some studies which described different results from ours [41–43]. This disagreement could be mainly explained by (1) different modalities of imaging used (Chest radiography *vs.* Ultrasonography *vs.* CT). Generally speaking, chest radiography is typically performed at the bedside (portable) in the setting of AP, and because of the improper positioning and image overlap, some effusions can be missed. When compared with chest radiography and CT, ultrasonography is dependent upon experience of the operator and is unsuitable for obese AP patients due to the echo attenuation. On the contrary, chest CT is extremely sensitive in detecting even minimal amounts of pleural effusion, and it can indicate infected pleural effusion (the so called “split pleura sign” on the chest CT [37]); (2) different number of AP patients were included among the studies and the patients’ clinical conditions varied largely. In this present study, we included the largest cohorts of AP patients (*n* = 465) and 222 (47.7%, 222/465) of them were diagnosed as moderately severe (*n* = 195, 41.9%, 195/465) or severe (*n* = 27, 5.8%, 27/465) AP.

Up to now, there is a lacking of a widely recognized grading system for pleural effusion quantification. However, several systems of classification of effusion size on chest radiography, ultrasonography, and CT have been proposed in the literature [44–47]. For example, in a study by Mironov et al. [45], on the basis of visual estimation, pleural effusion occupying less than one-third, one-third to two-thirds, and more than two-thirds of the visualized hemithorax were characterized as small, moderate, and large size, respectively. In another study, Moy et al. depicted a simple and two-step method for pleural effusions quantification on chest CT images by the usage a three-point grading standard [46]. As per their proposal, the first, second, and third or fourth antero-posterior quartile pleural effusions were defined as small, moderate, and large in size. For borderline cases, the upper limits of small and moderate pleural effusion were defined as the antero-posterior depth of 3.0 and 10.0 cm thresholds, respectively. When it comes to the mechanisms, several mechanisms have been described in the literature contributing to the formation of pleural effusion in patients with AP [37,48]. Of these, the transdiaphragmatic lymphatic blockage have been described the most [37]. There may also be a disruption of pancreatic duct which leads to the leakage of pancreatic enzymes and the formation of a pancreaticopleural fistula. The latter is more likely to occur if the duct disruption is posteriorly into the retroperitoneum. Exudation of fluid into the pleural cavity from the subpleural diaphragmatic vessels may also cause pleural effusion [37]. In addition, anatomy may also play an important role in the occurrence of pleural effusion in patients with AP. As there are some anatomical channels between the thoracic cavity and abdominal cavity, the inflammation of pancreas and surrounding areas may enter the thoracic cavity along these anatomical pathways [49].

In our study, we did not report the radiation dose for the following reasons: (1) relationship between radiation dose and AP is not the area of discussion in this study. Some publications have focussed on exploring the relationship between radiation dose and AP but more from the perspective of medical imaging technology rather than diagnostic view [50–52]. (2) since there is only limited data on the radiation dose from CT in patients with AP, it may be another potential topic in our future study.

Nevertheless, the authors still think that the radiation dose in our cohort of 465 AP patients was at an acceptable level (e.g. the U.S. Food and Drug Administration recommend that radiation dose for the chest and abdomen should be <7 and 8 mSv, respectively) [53,54]. At the authors’ hospital, a standard chest CT scanning range is from the lung apex to the bilateral costophrenic angle; and for standard upper abdominal CT scan, it is from diaphragmatic dome to bilateral iliac crest. As a result, if a separate standard chest and upper abdominal CT scan is performed for an individual, there will be some overlapping coverage, which can definitely increase the radiation dose. By contrast, the unenhanced chest and upper abdominal CT scan in our 465 AP patients were performed in one examination. Therefore, the overlapping coverage is avoided which would obviously decrease the radiation dose. In addition, we also applied novel techniques (e.g. SmartmA, CARE Dose 4D, Iterative Reconstruction) to reduce radiation during CT scan. These radiation reduction techniques have been shown to decrease the radiation dose while maintaining the image quality [55–57].

While interpreting the outcomes of this present study, some limitations should be clarified as well. First, retrospective nature of this present study may decrease its power of convincingness. Second, only AP patients with complete early thoracic and abdominal CT examinations were enrolled which may introduce a selection bias to this study. Third, there were variable intervals between the CT examinations and the onset of AP which may have an effect on the accuracy of CTSI and EPIC scores. Finally, we did not differentiate between moderately severe and severe AP, neither between transient nor persistent organ failure for evaluation. Nevertheless, no benefit has been shown to different treatment of these AP patients [15,17]. In addition, this present study was a multi-institutional one from three AP centres, and a complete comparison had also been performed between PEV and CRP levels, and Ranson, BISAP, Marshall, APACHE II, CTSI, and EPIC scoring systems. As a result, all the above mentioned limitations of this present study would have a little influence on its main conclusions.

## Conclusion

All in all, pleural effusion is a common chest finding in patients with acute pancreatitis. Pleural effusion volume quantified on chest CT examination positively associated with the duration of hospitalization, CRP levels, as well as Ranson, BISAP, Marshall, APACHE II, CTSI, and EPIC scoring systems. Pleural effusion volume can be a reliable radiologic biomarker in the prediction of severity and clinical outcomes of acute pancreatitis.

## Data Availability

The data related to the results of this study can be obtained from the first or corresponding author (GWY or YML) upon reasonable request.

## References

[1] Lankisch PG, Apte M, Banks PA. Acute pancreatitis. Lancet. 2015;386(9988):85–96. 25616312 10.1016/S0140-6736(14)60649-8

[2] Forsmark CE, Vege SS, Wilcox CM. Acute pancreatitis. N Engl J Med. 2016;375(20):1972–1981. 27959604 10.1056/NEJMra1505202 PMC13220086

[3] Majidi S, Golembioski A, Wilson SL, et al. Acute pancreatitis: etiology, pathology, diagnosis, and treatment. South Med J. 2017;110(11):727–732. 29100225 10.14423/SMJ.0000000000000727

[4] Wang GJ, Gao CF, Wei D, et al. Acute pancreatitis: etiology and common pathogenesis. World J Gastroenterol. 2009;15(12):1427–1430. 19322914 10.3748/wjg.15.1427 PMC2665136

[5] Zhu Y, Pan X, Zeng H, et al. A study on the etiology, severity, and mortality of 3260 patients with acute pancreatitis according to the revised Atlanta classification in Jiangxi, China over an 8-year period. Pancreas. 2017;46(4):504–509. 28196012 10.1097/MPA.0000000000000776

[6] Roberts SE, Morrison-Rees S, John A, et al. The incidence and aetiology of acute pancreatitis across Europe. Pancreatology. 2017;17(2):155–165. 28159463 10.1016/j.pan.2017.01.005

[7] Yang DD, Zuo HD, Wu CQ, et al. The characteristics of acute necrotizing pancreatitis in different age stages: an MRI study. Eur J Radiol. 2020;122:108752. 31778965 10.1016/j.ejrad.2019.108752

[8] Bradley EL III. A clinically based classification system for acute pancreatitis. Summary of the international symposium on acute pancreatitis, Atlanta, GA, September 11 through 13, 1992. Arch Surg. 1993;128(5):586–590. 8489394 10.1001/archsurg.1993.01420170122019

[9] Banks PA, Bollen TL, Dervenis C, et al. Classification of acute pancreatitis-2012: revision of the Atlanta classification and definitions by international consensus. Gut. 2013;62(1):102–111. 23100216 10.1136/gutjnl-2012-302779

[10] Thoeni RF. The revised Atlanta classification of acute pancreatitis: its importance for the radiologist and its effect on treatment. Radiology. 2012;262(3):751–764. 22357880 10.1148/radiol.11110947

[11] Zaheer A, Singh VK, Qureshi RO, et al. The revised Atlanta classification for acute pancreatitis: updates in imaging terminology and guidelines. Abdom Imaging. 2013;38(1):125–136. 22584543 10.1007/s00261-012-9908-0

[12] Zhao K, Adam SZ, Keswani RN, et al. Acute pancreatitis: revised Atlanta classification and the role of cross-sectional imaging. Am J Roentgenol. 2015;205(1):W32–W41. 26102416 10.2214/AJR.14.14056

[13] Foster BR, Jensen KK, Bakis G, et al. Revised Atlanta classification for acute pancreatitis: a pictorial essay. Radiographics. 2016;36(3):675–687. 27163588 10.1148/rg.2016150097

[14] Case BM, Jensen KK, Bakis G, et al. Endoscopic interventions in acute pancreatitis: what the advanced endoscopist wants to know. Radiographics. 2018;38(7):2002–2018. 30265612 10.1148/rg.2018180066

[15] Meyrignac O, Lagarde S, Bournet B, et al. Acute pancreatitis: extra pancreatic necrosis volume as early predictor of severity. Radiology. 2015;276(1):119–128. 25642743 10.1148/radiol.15141494

[16] del Val A, Pamies J, Collado JJ, et al. Extrapancreatic necrosis volume as early predictor of severity in acute pancreatitis. Pancreatology. 2017;17(5):S8.

[17] Çakar İ, Keven A, Eseroğlu E, et al. Role of extrapancreatic necrosis volume in determining early prognosis in patients with acute pancreatitis. Abdom Radiol. 2020;45(5):1507–1516. 10.1007/s00261-019-02188-9 31428812

[18] Pamies-Guilabert J, Del Val Antoñana A, Collado JJ, et al. Pancreatic necrosis volume – a new imaging biomarker of acute pancreatitis severity. Eur J Radiol. 2020;130:109193. 32768889 10.1016/j.ejrad.2020.109193

[19] Hameed AM, Lam VW, Pleass HC. Significant elevations of serum lipase not caused by pancreatitis: a systematic review. HPB. 2015;17(2):99–112. 24888393 10.1111/hpb.12277 PMC4299384

[20] Jany B, Welte T. Pleural effusion in adults-etiology, diagnosis, and treatment. Dtsch Arztebl Int. 2019;116(21):377–386. 31315808 10.3238/arztebl.2019.0377 PMC6647819

[21] Balthazar EJ, Robinson DL, Megibow AJ, et al. Acute pancreatitis: value of CT in establishing prognosis. Radiology. 1990;174(2):331–336. 2296641 10.1148/radiology.174.2.2296641

[22] De Waele JJ, Delrue L, Hoste EA, et al. Extrapancreatic inflammation on abdominal computed tomography as an early predictor of disease severity in acute pancreatitis: evaluation of a new scoring system. Pancreas. 2007;34(2):185–190. 17312456 10.1097/mpa.0b013e31802d4136

[23] Marshall JC, Cook DJ, Christou NV, Bernard GR, et al. Multiple organ dysfunction score: a reliable descriptor of a complex clinical outcome. Crit Care Med. 1995;23(10):1638–1652. 7587228 10.1097/00003246-199510000-00007

[24] Ranson JH, Rifkind KM, Roses DF, et al. Prognostic signs and the role of operative management in acute pancreatitis. Surg Gynecol Obstet. 1974;139(1):69–81. 4834279

[25] Wu BU, Johannes RS, Sun X, et al. The early prediction of mortality in acute pancreatitis: a large population-based study. Gut. 2008;57(12):1698–1703. 18519429 10.1136/gut.2008.152702

[26] Larvin M, McMahon MJ. APACHE-II score for assessment and monitoring of acute pancreatitis. Lancet. 1989;2(8656):201–205. 2568529 10.1016/s0140-6736(89)90381-4

[27] Koo TK, Li MY. A guideline of selecting and reporting intra class correlation coefficients for reliability research. J Chiropr Med. 2016;15(2):155–163. 27330520 10.1016/j.jcm.2016.02.012 PMC4913118

[28] Peng R, Zhang L, Zhang ZM, et al. Chest computed tomography semi-quantitative pleural effusion and pulmonary consolidation are early predictors of acute pancreatitis severity. Quant Imaging Med Surg. 2020;10(2):451–463. 32190570 10.21037/qims.2019.12.14 PMC7063295

[29] DeLong ER, DeLong DM, Clarke-Pearson DL. Comparing the areas under two or more correlated receiver operating characteristic curves: a nonparametric approach. Biometrics. 1988;44(3):837–845. 3203132

[30] Papachristou GI, Muddana V, Yadav D, et al. Comparison of BISAP, Ranson's, APACHE-II, and CTSI scores in predicting organ failure, complications, and mortality in acute pancreatitis. Am J Gastroenterol. 2010;105(2):435–441; quiz 442. 19861954 10.1038/ajg.2009.622

[31] Zhang J, Shahbaz M, Fang R, et al. Comparison of the BISAP scores for predicting the severity of acute pancreatitis in Chinese patients according to the latest Atlanta classification. J Hepatobiliary Pancreat Sci. 2014;21(9):689–694. 24850587 10.1002/jhbp.118

[32] Gao W, Yang HX, Ma CE. The value of BISAP score for predicting mortality and severity in acute pancreatitis: a systematic review and meta-analysis. PLOS One. 2015;10(6):e0130412. 26091293 10.1371/journal.pone.0130412 PMC4474919

[33] Harshit Kumar A, Singh Griwan M. A comparison of APACHE II, BISAP, Ranson's score and modified CTSI in predicting the severity of acute pancreatitis based on the 2012 revised Atlanta classification. Gastroenterol Rep. 2018; 6(2):127–131. 10.1093/gastro/gox029 PMC5952961 29780601

[34] Komolafe O, Pereira SP, Davidson BR, et al. Serum C-reactive protein, procalcitonin, and lactate dehydrogenase for the diagnosis of pancreatic necrosis. Cochrane Database Syst Rev. 2017;4(4):CD012645. 28431197 10.1002/14651858.CD012645 PMC6478063

[35] Li M, Xing XK, Lu ZH, et al. Comparison of scoring systems in predicting severity and prognosis of hypertriglyceridemia-induced acute pancreatitis. Dig Dis Sci. 2020;65(4):1206–1211. 31515723 10.1007/s10620-019-05827-9

[36] Gezer NS, Bengi G, Baran A, et al. Comparison of radiological scoring systems, clinical scores, neutrophil-lymphocyte ratio and serum C-reactive protein level for severity and mortality in acute pancreatitis. Rev Assoc Med Bras. 2020;66(6):762–770. 32696885 10.1590/1806-9282.66.6.762

[37] Kumar P, Gupta P, Rana S. Thoracic complications of pancreatitis. JGH Open. 2019;3(1):71–79. 30834344 10.1002/jgh3.12099 PMC6386740

[38] Raghu MG, Wig JD, Kochhar R, et al. Lung complications in acute pancreatitis. JOP. 2007;8(2):177–185. 17356240

[39] Liu D, Song B, Huang ZX, et al. The value of chest CT features evaluating the severity and prognosis for acute pancreatitis. Sichuan Da Xue Bao Yi Xue Ban. 2013;44(2):319–322. 23745281

[40] Raghuwanshi S, Gupta R, Vyas MM, et al. CT evaluation of acute pancreatitis and its prognostic correlation with CT severity index. J Clin Diagn Res. 2016;10(6):TC06–TC11. 10.7860/JCDR/2016/19849.7934 PMC4963736 27504376

[41] Maringhini A, Ciambra M, Patti R, et al. Ascites, pleural, and pericardial effusions in acute pancreatitis. A prospective study of incidence, natural history, and prognostic role. Dig Dis Sci. 1996;41(5):848–852. 8625753 10.1007/BF02091521

[42] Heller SJ, Noordhoek E, Tenner SM, et al. Pleural effusion as a predictor of severity in acute pancreatitis. Pancreas. 1997;15(3):222–225. 9336784 10.1097/00006676-199710000-00002

[43] Mergo PJ, Helmberger T, Didovic J, et al. New formula for quantification of pleural effusions from computed tomography. J Thorac Imaging. 1999;14(2):122–125. 10210486 10.1097/00005382-199904000-00011

[44] Ocampo C, Silva W, Zandalazini H, et al. Pleural effusion is superior to multiple factor scoring system in predicting acute pancreatitis outcome. Acta Gastroenterol Latinoam. 2008;38(1):34–42. 18533355

[45] Mironov O, Ishill NM, Mironov S, et al. Pleural effusion detected at CT prior to primary cytoreduction for stage III or IV ovarian carcinoma: effect on survival. Radiology. 2011;258(3):776–784. 21193598 10.1148/radiol.10100162 PMC3713162

[46] Moy MP, Levsky JM, Berko NS, et al. A new, simple method for estimating pleural effusion size on CT scans. Chest. 2013;143(4):1054–1059. 23632863 10.1378/chest.12-1292 PMC3616681

[47] Teichgräber UK, Hackbarth J. Sonographic bedside quantification of pleural effusion compared to computed tomography volumetry in ICU patients. Ultrasound Int Open. 2018;4(4):E131–E135. 30374471 10.1055/a-0747-6416 PMC6203686

[48] Uchiyama T, Suzuki T, Adachi A, et al. Pancreatic pleural effusion: case report and review of 113 cases in Japan. Am J Gastroenterol. 1992;87(3):387–391. 1539580

[49] Xu H, Ebner L, Jiang S, et al. Retrocrural space involvement on computed tomography as a predictor of mortality and disease severity in acute pancreatitis. PLOS One. 2014;9(9):e107378. 25222846 10.1371/journal.pone.0107378 PMC4164622

[50] Gupta P, Jain R, Koshi S, et al. Radiation dose from computed tomography in patients with acute pancreatitis: an audit from a tertiary care referral hospital. Abdom Radiol. 2020;45(5):1517–1523. 10.1007/s00261-020-02408-7 31960118

[51] Leung VJ, Godfrey EM, Biddle DJ, et al. Split-bolus single-pass CT for vascular complications in acute pancreatitis: assessment of radiation dose and multi-phasic contrast enhancement compared to single-bolus multi-pass CT. Clin Radiol. 2020;75(8):644.e1–644.e6. 10.1016/j.crad.2020.05.002 32560906

[52] Avanesov M, Weinrich JM, Kraus T, et al. MDCT of acute pancreatitis: intraindividual comparison of single-phase versus dual-phase MDCT for initial assessment of acute pancreatitis using different CT scoring systems. Eur J Radiol. 2016;85(11):2014–2022. 27776654 10.1016/j.ejrad.2016.09.013

[53] Available from: https://www.fda.gov/radiation-emitting-products/medical-x-ray-imaging/what-are-radiation-risks-ct

[54] McCollough CH, Bushberg JT, Fletcher JG, et al. Answers to common questions about the use and safety of CT scans. Mayo Clin Proc. 2015;90(10):1380–1392. 26434964 10.1016/j.mayocp.2015.07.011

[55] Wang L, Gong S, Yang J, et al. CARE dose 4D combined with sinogram-affirmed iterative reconstruction improved the image quality and reduced the radiation dose in low dose CT of the small intestine. J Appl Clin Med Phys. 2019;20(1):293–307. 30508275 10.1002/acm2.12502 PMC6333130

[56] Ye K, Chen M, Li J, et al. Ultra-low-dose CT reconstructed with Asir-V using SmartmA for pulmonary nodule detection and Lung-RADS classifications compared with low-dose CT. Clin Radiol. 2021;76(2):156.e1–156.e8. 10.1016/j.crad.2020.10.014 33293025

[57] Zhu Z, Zhao Y, Zhao X, et al. Impact of preset and postset adaptive statistical iterative reconstruction-V on image quality in nonenhanced abdominal-pelvic CT on wide-detector revolution CT. Quant Imaging Med Surg. 2021;11(1):264–275. 33392027 10.21037/qims-19-945 PMC7719942

